# Correction: Microscopic distance from tumor invasion front to serosa might be a useful predictive factor for peritoneal recurrence after curative resection of T3-gastric cancer

**DOI:** 10.1371/journal.pone.0233743

**Published:** 2020-05-26

**Authors:** Shingo Togano, Masakazu Yashiro, Yuichiro Miki, Yurie Yamamoto, Tomohiro Sera, Yukako Kushitani, Atsushi Sugimoto, Shuhei Kushiyama, Sadaaki Nishimura, Kenji Kuroda, Tomohisa Okuno, Mami Yoshii, Tatsuro Tamura, Takahiro Toyokawa, Hiroaki Tanaka, Kazuya Muguruma, Sayaka Tanaka, Masaichi Ohira

The fourth author's name is spelled incorrectly. The correct name is: Yurie Yamamoto. The correct citation is: Togano S, Yashiro M, Miki Y, Yamamoto Y, Sera T, Kushitani Y, et al. (2020) Microscopic distance from tumor invasion front to serosa might be a useful predictive factor for peritoneal recurrence after curative resection of T3-gastric cancer. PLoS ONE 15(1): e0225958. https://doi.org/10.1371/journal.pone.0225958
