# Supplementary material for: Microscopic distance from tumor invasion front to serosa might be a useful predictive factor for peritoneal recurrence after curative resection of T3-gastric cancer
Source: PLoS One. 2020 Jan 15;15(1):e0225958. doi: 10.1371/journal.pone.0225958 (PMC6961828; doi:10.1371/journal.pone.0225958)
Supplement: S1 Table — (DOCX) [file pone.0225958.s001.docx]

**S1 Table.** **Correlation between peritoneal recurrence and clinicopathologic features in 96 gastric cancer cases at T3 stage.**

| Clinicopathologic features | Peritoneal recurrence (n=16) | No peritoneal recurrence (n=80) | p value |
| --- | --- | --- | --- |
| Tumor location  Upper third  Middle third  Lower third | 4 (11.8%)  4 (13.3%)  8 (25.0%) | 30 (88.2%)  26 (86.7%)  24 (75.0%) | 0.297 |
| Tumor location  Anterior wall  Posterior wall  Greater curvature  Lesser curvature  Circumferential | 1 (10.0%)  2 (11.1%)  3 (30.0%)  9 (17.0%)  1 (20.0%) | 9 (90.0%)  16 (88.9%)  7 (70.0%)  44 (83.0%)  4 (80.0%) | 0.728 |
| Method of resection  Distal gastrectomy  Total gastrectomy | 10 (17.9%)  6 (15.0%) | 46 (82.1%)  34 (85.0%) | 0.787 |
| Extent of LN dissection^a^  D  D1+  D2 | 2 (15.4%)  1 (12.5%)  13 (17.3%) | 11 (84.6%)  7 (87.5%)  62 (82.7%) | 0.933 |
| Adjuvant chemotherapy  S-1^b^  UFT^c^  Doxifluridine  None | 4 (14.8%)  7 (21.9%)  1 (20.0%)  4 (12.5%) | 23 (85.2%)  25 (78.1%)  4 (80.0%)  28 (87.5%) | 0.769 |

^a^: LN dissection ; Lymph node dissection

^b^: S-1 ; Tegafur-gimeracil-oteracil

^c^: UFT ; Uracil and tegafur
