# Supplementary material for: Microscopic distance from tumor invasion front to serosa might be a useful predictive factor for peritoneal recurrence after curative resection of T3-gastric cancer
Source: PLoS One. 2020 Jan 15;15(1):e0225958. doi: 10.1371/journal.pone.0225958 (PMC6961828; doi:10.1371/journal.pone.0225958)
Supplement: S2 Table — (DOCX) [file pone.0225958.s002.docx]

**S2 Table. Correlation between DIFS ≤234 and lymph node metastasis.**

| **Clinicopathologic feature** | **DIFS^a^ < 234 µm (n=58)** | **DIFS > 234 µm (n=38)** | **p value** |
| --- | --- | --- | --- |
| LN metastasis^b^  negative  positive | 25 (52.1%)  33 (68.8%) | 23 (47.9%)  15 (31.2%) | 0.095 |

^a^: DIFS; the microscopic distance from tumor invasion front to serosa

^b^: LN metastasis; Lymph node metastasis
