# Supplementary material for: Microscopic distance from tumor invasion front to serosa might be a useful predictive factor for peritoneal recurrence after curative resection of T3-gastric cancer
Source: PLoS One. 2020 Jan 15;15(1):e0225958. doi: 10.1371/journal.pone.0225958 (PMC6961828; doi:10.1371/journal.pone.0225958)
Supplement: Attachment — Submitted filename: Response to Reviewers.docx [file pone.0225958.s003.docx]

**Reviewer #1**

Thank you very much for the careful review of the Reviewer #1. We corrected several points according to the descriptions by the Reviewer #1, as described below. We indicated the changes point by point and highlighted them in the revised paper.

1. This study evaluated only T3-gastric cancer.

I suggest to change the title. "gastric cancer" to "T3-gastric cancer"

According to the reviewer#1's comment, we change the title, "Microscopic distance from tumor invasion front to serosa might be a useful predictive factor for peritoneal recurrence after curative resection of T3-gastric caner". (on page 1, line 3)

2. Why did the authors focus on the microscopic distance from the tumor invasion front to the serosa (DIFS) and E-cadherin. Please mentioned in Introduction section.

It has been reported that E-cadherin is one of important factors for tumor invasion and distant metastasis in some solid cancers [8, 10-12]. Taken together, we previously reported the correlation between DIFS and serosal exposure of gastric cancer cells, and speculated that DIFS might be associated with peritoneal recurrence [3]. Then, in this study we focused on the significance of DIFS and E-cadherin in peritoneal recurrence. We added the comments in the introduction. (on page 3 line 40-45)

[3] Miki Y, Yashiro M, Ando K, Okuno T, Kitayama K, Masuda G, et al. Examination of cancer cells exposed to gastric serosa by serosal stamp cytology plus RT-PCR is useful for the identification of gastric cancer patients at high risk of peritoneal recurrence. Surgical oncology. 2017;26(4):352-8. Epub 2017/11/09. doi: 10.1016/j.suronc.2017.07.008. PubMed PMID: 29113652.

[8] Sun F, Feng M, Guan W. Mechanisms of peritoneal dissemination in gastric cancer. Oncology letters. 2017;14(6):6991-8. Epub 2018/01/19. doi: 10.3892/ol.2017.7149. PubMed PMID: 29344127; PubMed Central PMCID: PMCPMC5754894.

[10] Gao M, Zhang X, Li D, He P, Tian W, Zeng B. Expression analysis and clinical significance of eIF4E, VEGF-C, E-cadherin and MMP-2 in colorectal adenocarcinoma. Oncotarget. 2016;7(51):85502-14. Epub 2016/12/03. doi: 10.18632/oncotarget.13453. PubMed PMID: 27907907; PubMed Central PMCID: PMCPMC5356753.

[11] Liang G, Ding M, Lu H, Cao NA, Niu Y, Gao Y, et al. Metformin upregulates E-cadherin and inhibits B16F10 cell motility, invasion and migration. Oncology letters. 2015;10(3):1527-32. Epub 2015/12/02. doi: 10.3892/ol.2015.3475. PubMed PMID: 26622703; PubMed Central PMCID: PMCPMC4533732.

[12] Torabizadeh Z, Nosrati A, Sajadi Saravi SN, Yazdani Charati J, Janbabai G. Evaluation of E-cadherin Expression in Gastric Cancer and Its Correlation with Clinicopathologic Parameters. International journal of hematology-oncology and stem cell research. 2017;11(2):158-64. Epub 2017/09/07. PubMed PMID: 28875011; PubMed Central PMCID: PMCPMC5575728.

3. Please clarify the inclusion criteria. The authors should describe the study period and flow chart.

The inclusion criteria were as follows; 1. histologically proven gastric adenocarcinoma; 2. the depth of tumor invasion was T3; 3. curative operation; 4. intraoperative peritoneal lavage cytology-negative. The follow-up period was 60 months, and the median follow-up was 49.3 months. We described these criteria in the materials and Fig. 1. (on page 4 line 52-58)

4. The authors have used the UICC/AJCC 7th edition staging manual. Please use the most current staging manual.

We changed the 7th edition of TNM Classification to the 8th edition of TNM Classification [15]. (on page 5 line 71)

[15] Brierley JD, Gospodarowicz MK, Writtekind C. TNM Classification of Malignant Tumours, 8th Edition 2016.

5. As the authors mentioned in Introduction section, we sometimes experienced peritoneal recurrence even in T1-2 cancer patients. Why T1-2 cancer was not included in this analysis? They should include T1-2 cancers in this study.

Since the peritoneal recurrence of T1 and T2 cancers has been considered to develop via trans-lymphatic pathway [13, 14], we excluded T1 and T2 cases in this study. (on page 4 line 55-57)

[13] Yoshida M, Sugino T, Kusafuka K, Nakajima T, Makuuchi R, Tokunaga M, et al. Peritoneal dissemination in early gastric cancer: importance of the lymphatic route. Virchows Arch. 2016;469(2):155-61. Epub 2016/05/26. doi: 10.1007/s00428-016-1960-7. PubMed PMID: 27220762.

[14] Yamamoto M, Taguchi K, Baba H, Endo K, Kohnoe S, Okamura T, et al. Peritoneal dissemination of early gastric cancer: report of a case. Surgery today. 2006;36(9):835-8. Epub 2006/08/29.

6. In this analysis, location of the tumor (Upper/ Middle/ Lower and Anterior/ Posterior/ Grater curvature side/ Lesser curvature side) was not included. These factors should be included.

We included the tumor location in S1 Table.

**Reviewer #2**

Thank you very much for the careful review of the Reviewer #2. We correct several points according to the descriptions by the Reviewer #2, as follows.

1. Did the authors examine peritoneal washing cytology? If so, the authors should describe the result and analyze the relationship between peritoneal recurrence and cytology.

We performed peritoneal washing cytology in all cases. Peritoneal washing cytology-positive cases were excluded in this study, which was described in the materials and Fig. 1. (on page 4 line 52-55)

2. The authors should show the clinicopathological features in more detail in Table 1, including tumor location, type of gastrectomy, extent of lymph node dissection, and the number of the patients who underwent adjuvant chemotherapy.

We added the detail of clinicopathological features, including tumor location, type of gastrectomy, extent of lymph node dissection, and the number of the patients who underwent adjuvant chemotherapy in S1 Table.

3. In Material and methods section (page 5, line 77-82), the author should describe who evaluated E-cadherin IHC staining (e.g. by experienced pathologist unaware of clinical data). If possible, the authors should perform the agreement study of E-cadherin expression and show the concordance between the two pathologist.

The pathologist, Dr Sayaka Tanaka, checked DIFS and E-cadherin expression. Dr Togano S, Dr Yashiro M, and Dr Tanaka S checked DIFS and E-cadherin expression. We added these comments in the materials and methods. (on page 6 line 119-121)

4. The authors should discuss several limitations of this study in Discussion section.

We added limitations in the manuscript, as follows. Since it is difficult to examine the whole lesions of tumor, it is uncertain that the obtained section represented most invasive lesion of cancer cells. (on page 14 line 347-349)

5. Please recheck the grammar and terminology.

The manuscript was proofread by a professional editor and native English speaker.

6. The statistical method for the multivariate analysis is unclear.

Multivariate analysis with respect to peritoneal recurrence was performed using logistic regression analysis. Multivariate analysis with respect to five-year overall survival was performed using Cox proportional hazard model. Covariates were selected from those with significant differences in univariate analysis. (on page 7 line 130-133)

**Reviewer #3**

Thank you very much for the careful review of the Reviewer #3. We correct several points according to the descriptions by the Reviewer #3, as follows.

1. The authors focused on the risk factors of peritoneal metastasis after curative resection for T3 gastric cancer. The title does not include the information that this study especially focused on T3 gastric cancer.

According to the reviewer's comments, we changed the title, "Microscopic distance from tumor invasion front to serosa might be a useful predictive factor for peritoneal recurrence after curative resection of T3-gastric cancer" (on page 1, line 3)

2. The follow-up period of the study cohort is very important information for readers. Besides, it is also important how these patients were followed up. The authors should provide the information on the follow-up period, timing and modality for follow-up.

We added follow-up period, timing and modality in Materials and Method section, as follows. The follow-up period was 60 months, and the median follow-up was 49.3 months. The follow-up program of postoperative surveillance consisted of computed tomography, and ultrasound performed every 3 months in order to diagnose recurrent diseases. (on page 4 line 57-60)

3. Adjuvant chemotherapy may influence the survival of patients. Please include the information on presence or absence of adjuvant chemotherapy into the background.

We included the data of adjuvant chemotherapy in S1 Table.

4. When discussing the DIFS, the preparation of tissue section for pathology is very important information. Please provide the details of preparation for tissue section.

The preparation of tissue section was as follows. After gastrectomy, the gastric tumor was immediately treated with 10% formalin neutral buffer solution for 24-72 hours. We added the comments in Materials and Method section. (on page 5 line 79-80)

5. Please provide the C-statistic of the ROC curve shown in Figure 2.

We added the C-statistic (0.66) in Figure 2.

6. The authors should describe the detail of multivariate analysis. How did they select the covariates?

Multivariate analysis with respect to peritoneal recurrence was performed using logistic regression analysis. Multivariate analysis with respect to five-year overall survival was performed using Cox proportional hazard model. Covariates were selected from those with significant differences in univariate analysis. (on page 7 line 130-133)

7. The discussion seems to be poor. Please discuss why the expression of E-Cadherin did not influence the peritoneal metastasis in this study cohort, and the clinical evidence of adjuvant therapy to decrease peritoneal recurrence. Also, they should declare the limitation of this study.

One of the reasons why E-cadherin did not affect the peritoneal recurrence of T3 cases might be that E-cadherin might affect the invasion activity of cancer cells at early T-stage such as T1 and T2, but not advanced T-stage such as T3 and T4. (on page 13 line 334- page 13 line 340)

According to guideline, tegafur-gimeracil-oteracil (TS-1) monotherapy is recommended for the gastric cancer patients with stage II, and oxaliplatin combination therapy is recommended for the gastric cancer patients with stage III in Japan [2.17-19]. Our study might suggest that oxaliplatin combination therapy may be recommended not only for stage III but also for stage II with DIFS ≤ 234 µm. (on page 13 line 317-321).

We added limitations in the manuscript, as follows. Since it is difficult to examine the whole lesions of tumor, it is uncertain that the obtained section represented most invasive lesion of cancer cells. (on page 14 line 348-350).

[2] Sakuramoto S, Sasako M, Yamaguchi T, Kinoshita T, Fujii M, Nashimoto A, et al. Adjuvant chemotherapy for gastric cancer with S-1, an oral fluoropyrimidine. The New England journal of medicine. 2007;357(18):1810-20. Epub 2007/11/06. doi: 10.1056/NEJMoa072252. PubMed PMID: 17978289.

[17] Sasako M, Sakuramoto S, Katai H, Kinoshita T, Furukawa H, Yamaguchi T, et al. Five-year outcomes of a randomized phase III trial comparing adjuvant chemotherapy with S-1 versus surgery alone in stage II or III gastric cancer. Journal of clinical oncology : official journal of the American Society of Clinical Oncology. 2011;29(33):4387-93. Epub 2011/10/20. doi: 10.1200/jco.2011.36.5908. PubMed PMID: 22010012.

[18] Bang YJ, Kim YW, Yang HK, Chung HC, Park YK, Lee KH, et al. Adjuvant capecitabine and oxaliplatin for gastric cancer after D2 gastrectomy (CLASSIC): a phase 3 open-label, randomised controlled trial. Lancet (London, England). 2012;379(9813):315-21. Epub 2012/01/10. doi: 10.1016/s0140-6736(11)61873-4. PubMed PMID: 22226517.

[19] Noh SH, Park SR, Yang HK, Chung HC, Chung IJ, Kim SW, et al. Adjuvant capecitabine plus oxaliplatin for gastric cancer after D2 gastrectomy (CLASSIC): 5-year follow-up of an open-label, randomised phase 3 trial. The Lancet Oncology. 2014;15(12):1389-96. Epub 2014/12/03. doi: 10.1016/s1470-2045(14)70473-5. PubMed PMID: 25439693.

**Reviewer #4**

Thank you very much for the careful review of the Reviewer #4. We correct several points according to the descriptions by the Reviewer #4, as follows.

Major points

1. DIFS is very important in this article. So they should describe the precise method to measure the DIFS. For example, how many did they check to confirm the invasion front. They should clarify the identification of DIFS in details.

We checked invasion depth of all specimens of the tumor using H-E staining slides. After selecting three specimens with high invasion depth, the three specimens were stained by Pan-cytokeratin and measured DIFS of 3 slides. The shortest distance was defined as DIFS of the case. We added the comments in Materials and Method section. (on page 6 line 108-111)

2. They classified the patients to peritoneal recurrence and non-recurrence groups. How long did they observe those patients? They should describe the observation period.

The follow-up period was 60 months, and the median follow-up was 49.3 months. We added follow-up period in Materials and Method section. (on page 4 line 57-58)

3. The explanation of the importance of E-cadherin in gastric cancer patients is necessary.

It has been reported that E-cadherin is one of important factors for tumor invasion and distant metastasis in some solid cancers [8, 10-12]. We added the comments in the introduction. (on page 3 line 40-45)

[8] Sun F, Feng M, Guan W. Mechanisms of peritoneal dissemination in gastric cancer. Oncology letters. 2017;14(6):6991-8. Epub 2018/01/19. doi: 10.3892/ol.2017.7149. PubMed PMID: 29344127; PubMed Central PMCID: PMCPMC5754894.

[10] Gao M, Zhang X, Li D, He P, Tian W, Zeng B. Expression analysis and clinical significance of eIF4E, VEGF-C, E-cadherin and MMP-2 in colorectal adenocarcinoma. Oncotarget. 2016;7(51):85502-14. Epub 2016/12/03. doi: 10.18632/oncotarget.13453. PubMed PMID: 27907907; PubMed Central PMCID: PMCPMC5356753.

[11] Liang G, Ding M, Lu H, Cao NA, Niu Y, Gao Y, et al. Metformin upregulates E-cadherin and inhibits B16F10 cell motility, invasion and migration. Oncology letters. 2015;10(3):1527-32. Epub 2015/12/02. doi: 10.3892/ol.2015.3475. PubMed PMID: 26622703; PubMed Central PMCID: PMCPMC4533732.

[12] Torabizadeh Z, Nosrati A, Sajadi Saravi SN, Yazdani Charati J, Janbabai G. Evaluation of E-cadherin Expression in Gastric Cancer and Its Correlation with Clinicopathologic Parameters. International journal of hematology-oncology and stem cell research. 2017;11(2):158-64. Epub 2017/09/07. PubMed PMID: 28875011; PubMed Central PMCID: PMCPMC5575728.

4. DIFS and lymph node metastasis are independent predictive factors for peritoneal recurrence. So they should add subgroup analysis in T3, lymph node metastasis negative patients.

We performed subgroup analysis for lymph node metastasis negative patients (n=48), positive patients (n=48). Only 3 cases developed in lymph node metastasis negative patients (n=48). There was no significant correlation between DIFS ≤234 µm and peritoneal recurrence (data not shown).

Minor points
1. In Table1, they should include DIFS and E-cadherin expression.
2. In line 189, “by H&E staining” is unnecessary.

According to the comment, we include DIFS and E-cadherin expression to Table 1 and omit a sentence, “by H&E staining” In line 189.

**Reviewer #5**

Thank you very much for the careful review of the Reviewer #5. We correct several points according to the descriptions by the Reviewer #5, as follows.

1. The authors measured the microscopic distance from the tumor invasion front to the serosa (DIFS) using tissue slides by H&E staining and pan-cytokeratin. However, there are no description about the numbers of slides they evaluated the DIFS. It might be necessary to measure at least 3 slides per case to evaluate DIFS precisely.

We checked invasion depth of all specimens of the tumor using H-E staining slides. After selecting three specimens with high invasion depth, the three specimens were stained by Pan-cytokeratin and measured DIFS of 3 slides. The shortest distance was defined as DIFS of the case. We added the comments in Materials and Method section. (on page 6 line 108-111)

2. The authors did not mention about the adjuvant chemotherapy. The authors should describe the presence or absence of adjuvant chemotherapy, and also indicate the contents of adjuvant chemotherapy.

We showed the data of adjuvant chemotherapy in S1 Table.

3. The authors pointed out that the lymph node metastasis were independent risk factors for peritoneal recurrence. And they assumed that the serosal invasion may occur via translymphatic pathway. It should be desireble to present the mechanism of serosal invasion may occur via translymphatic pathway. And they should evaluate the exposure of cancer cell at the surface of lymph node which the cancer cells metastases.

We commented as follows; peritoneal recurrence in gastric cancer without serosal invasion may occur via trans-lymphatic pathway. (on page 13 line 327-330)

Evaluation of the exposure of cancer cells from the lymph nodes may be useful to prove peritoneal recurrence via trans-lymphatic pathway. However, lymph node metastasis might be secondary developed after peritoneal recurrence. Therefore, it is difficult to evaluate whether peritoneal metastasis might occur via trans-lymphatic pathway or serosal exposure of cancer cells.
